# Modeling the measurement precision of Fringe Projection Profilometry

**DOI:** 10.1038/s41377-023-01294-0

**Published:** 2023-10-30

**Authors:** Shenzhen Lv, Qian Kemao

**Affiliations:** School of Computer Science and Engineering, Nanyang Technological University, Singapore, Singapore

**Keywords:** Imaging and sensing, Optical sensors

## Abstract

Three-dimensional (3D) surface geometry provides elemental information in various sciences and precision engineering. Fringe Projection Profilometry (FPP) is one of the most powerful non-contact (thus non-destructive) and non-interferometric (thus less restrictive) 3D measurement techniques, featuring at its high precision. However, the measurement precision of FPP is currently evaluated experimentally, lacking a complete theoretical model for guidance. We propose the first complete FPP precision model chain including four stage models (camera intensity, fringe intensity, phase and 3D geometry) and two transfer models (from fringe intensity to phase and from phase to 3D geometry). The most significant contributions include the adoption of a non-Gaussian camera noise model, which, for the first time, establishes the connection between camera’s electronics parameters (known in advance from the camera manufacturer) and the phase precision, and the formulation of the phase to geometry transfer, which makes the precision of the measured geometry representable in an explicit and concise form. As a result, we not only establish the full precision model of the 3D geometry to characterize the performance of an FPP system that has already been set up, but also explore the expression of the highest possible precision limit to guide the error distribution of an FPP system that is yet to build. Our theoretical models make FPP a more designable technique to meet the challenges from various measurement demands concerning different object sizes from macro to micro and requiring different measurement precisions from a few millimeters to a few micrometers.

## Graphical abstract

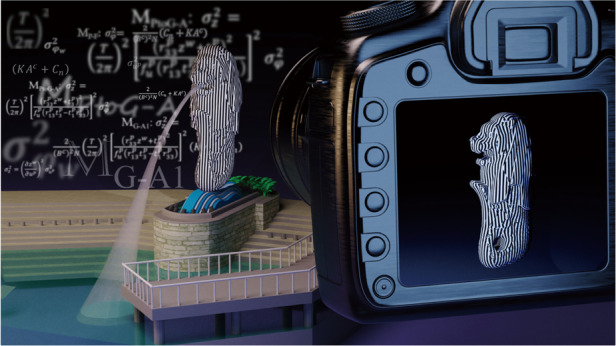

## Introduction

Three-dimensional (3D) surface geometry provides elemental information for the understanding of the world in the tasks such as self-driving vehicles^1^ and even for the augmenting of the world in metaverse.^2^ Many optical 3D shape measurement techniques have been developed, including LiDAR,^3^ stereo-vision,^4^ time of flight,^5^ speckle projection,^6^ and fringe projection profilometry (FPP).^7^ Among the non-interferometric techniques, FPP is outstanding for its merits of being non-contact, low-cost, high-speed and especially high-precision, and has been successfully applied in industrial manufacturing,^8^ robotics,^9^ spectral measurement,^10^ biomedicine,^11^ forensic science,^12^ etc. Having said that, it is of much surprise that there is a lack of a theoretical precision model for FPP, to the best of our knowledge, and the precision measure is often obtained experimentally.^13^ This gives two challenges in practice. First, it is difficult to predict the measurement precision and thus lacks theoretical guidance for the FPP system design; Second, although the precision can be measured, there is no theoretical-experimental cross verification. Developing a complete theoretical precision transfer model is thus highly demanded and is the motivation of this paper.

FPP systems includes one-camera and one-projector systems,^14^ fringe projection assisted multi-view systems^15^ and coaxial systems.^16^ Without loss of generality, this paper focuses on the analysis of the most widely adopted and extensively studied one-camera and one-projector system as a typical example, which can serve as good reference for the other two types of systems. A typical FPP includes a projector, a camera, and a computer, where the projector is used to project the fringes onto the surface of the object, the camera captures the deformed fringes, and the computer process the captured images, extracts the phase information, and reconstructs a 3D shape by a triangulation method. Accordingly, the measurement process includes a capturing step and the following three computing steps: (1) from the phase-shifted fringe patterns captured by the camera, phase is calculated; (2) with the calculated phase, the camera-projector pixel-correspondence is established; and (3) from the corresponding camera and projector pixels, 3D coordinate is reconstructed by triangulation.^17^ The last computing step requires the FPP system to be pre-calibrated which can be achieved by either directly establishing the relationship between the absolute phase distribution and the 3D information,^18–20^ or using the stereo-vision-based model proposed by Zhang et al. ^14^. The latter is adopted in this work due to it high accuracy, precision, and convenience. Although the calibration is extremely important, we argue that once the FPP system is calibrated, the calibration error will only affect the measurement accuracy, not the precision, and thus not considered for our precision analysis in this paper. Furthermore, in real measurement, there are error sources such as lens distortion,^21^ gamma effects,^22^ intensity saturation,^23^ etc., which can be corrected or mitigated.^17,21–24^ As they affect the measurement accuracy, they could also be calibrated.

**Fig. 1:**
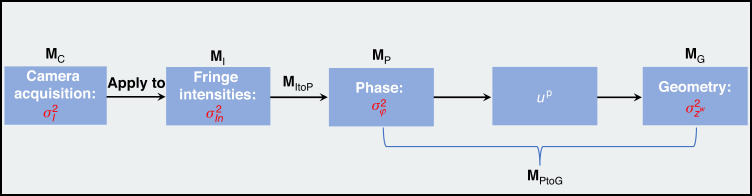
The full-chain noise models of the FPP system

From the above-mentioned measurement procedure, the random noise is generated in the capturing step and then transfers in the three computing steps, until it reaches the final reconstruction result. Such step-by-step measurement procedure enables us to propose our complete and generic noise model chain with the following four stage models and two transfer models: (S1) a model of camera noise (**M**_C_); (S2) a model of fringe intensity noise (**M**_I_) by applying **M**_C_ to fringe patterns; (S3) a model of phase noise (**M**_P_) which is important to evaluate the phase quality; (S4) a model of geometry noise (**M**_G_) which is important to evaluate the final geometry quality; (T1) a model to transfer noise from fringe intensity to phase (**M**_ItoP_); and (T2) a model to transfer noise from phase to the geometry (**M**_PtoG_). All these models are illustrated in Fig. 1, where the computing steps (2) and (3) are combined into **M**_PtoG_ for simplicity.

Although our model chain seems apparent, it has not been clearly described in the FPP community. Among all the individual models, our research focus will be on **M**_C_, **M**_P_, **M**_G_ and **M**_PtoG_, since **M**_I_ can be directly obtained from **M**_C_ while **M**_ItoP_ has been extensively studied.^25–28^ The problem statement and our contributions are as follows:

(i) For **M**_C_, the Gaussian model^25–27,29–31^ has been almost exhaustively used in the FPP community, because many noise distributions can be approximated as Gaussian, and when independent random variables are summed up, their properly normalized sum tends toward a normal distribution (central limit theorem).^32^ However, little experimental validation on the Gaussian model has been provided. In fact, there is another standard camera noise model^33^ which has been commonly used in Machine Vision field, but unfortunately, not yet been adopted in FPP, to the best of our knowledge. Although some observations on non-Gaussian noise have been made in,^34^ the noise treatment is rather empirical. Therefore, it is necessary to re-examine the camera noise modeling. We adopt this standard camera noise model into FPP for the first time. The most significant feature of the new model is that its parameters can be obtained from the manufacturer in advance without any measurement, making the precision prediction of FPP possible;
(ii) For **M**_P_, although the phase distribution is an intermediate variable, it is important for the evaluation of various techniques including fringe pattern design, phase-shifting technique, and phase unwrapping technique. We develop three phase noise models, from most complicated to simplest, for different purposes;
(iii) For **M**_PtoG_, in our previous work, we reported some initial results for the purpose of proving the equivalence of different measurement methods (OptE3, OptR4 and HorVer4) with respect to measurement precision, but not in a form of precision model nor in the pursuit of precision limit.^17^ In this paper, we derive an explicit expression and approximate it according to practical conditions. These results not only reveal the roles of the physical parameters in determining the measurement precision, but also effectively guide the design of FPP system.
(iv) For **M**_G_, since it is at the final stage of measurement, an integrated form will be derived to enable the characterization of the entire FPP system.

In summary, in this paper, all six individual models are established, and three key stage models, **M**_C_, **M**_P_, and **M**_G_, will be validated experimentally. The significance of our work is twofold: it provides a guide for the system configuration and hardware selection during system design with a given error tolerance and provides a theoretical basis for precision estimation after the system has been set up, which are important both theoretically and practically. In addition, our phase noise model can provide reference value to other optical methods and instruments, such as fringe projection assisted multi-view systems^15,35^ and coaxial systems,^16^ interferometry^36–38^ and deflectometry.^39,40^

The rest of this paper is arranged as follows: the basic principles of an FPP system are given in Principle of FPP; **M**_ItoP_ and **M**_PtoG_ models are described in Two transfer models of noise; all four proposed stage models are explained in Four stage models of noise; comprehensive experimental validation is given in Experimental verification; further discussions are provided in Discussions and the paper is concluded in Conclusion.

## Principle of FPP

In this section, the principle of FPP is briefly introduced, as a preparation for all the stage and transfer models.

### Pinhole models in an FPP system

Since FPP can be seen as a triangulation method for 3D reconstruction, the pinhole model is often used to describe the imaging process of the camera and the projector. The pinhole model of the camera can be expressed as,^17^

1 $${s}^{c}\left[\begin{array}{c}{u}^{c}\\ {v}^{c}\\ 1\end{array}\right]=\left[\begin{array}{ccc}{f}_{u}^{c} & 0 & {u}_{0}^{c}\\ 0 & {f}_{v}^{c} & {v}_{0}^{c}\\ 0 & 0 & 1\end{array}\right]\left[\begin{array}{ccc}{r}_{11}^{c} & {r}_{12}^{c} & \begin{array}{cc}{r}_{13}^{c} & {t}_{1}^{c}\end{array}\\ {r}_{21}^{c} & {r}_{22}^{c} & \begin{array}{cc}{r}_{23}^{c} & {t}_{2}^{c}\end{array}\\ {r}_{31}^{c} & {r}_{32}^{c} & \begin{array}{cc}{r}_{33}^{c} & {t}_{3}^{c}\end{array}\end{array}\right]\left[\begin{array}{c}{x}^{w}\\ {y}^{w}\\ \begin{array}{c}{z}^{w}\\ 1\end{array}\end{array}\right]$$

where the superscript *w* denotes the world coordinate system; $\left({x}^{w},{y}^{w},{z}^{w}\right)$ indicates the coordinate of the object point in the world coordinate system; the superscript *c* highlights that we are modeling the camera imaging system; $\left({u}^{c},{v}^{c}\right)$ indicates the coordinate of the image of an object point in the pixel coordinate system; ${f}_{u}^{c}$ and ${f}_{v}^{c}$ (unit: pixel) are the focal lengthes of the camera lens along ${u}^{c}$ and ${v}^{c}$ directions, respectively; $\left({u}_{0}^{c},{v}_{0}^{c}\right)$ is the camera’s principle point coordinate; ${s}^{c}$ is a scalar; ${r}_{{ij}}^{c}$ and ${t}_{j}^{c}$ $\left(i=\mathrm{1,2,3}{;j}=\mathrm{1,2,3}\right)$ are the entries of the rotation matrix ${{R}}^{c}$ and translation vector ${{\bf{t}}}^{c}$, respectively, to describe the transformation from the world coordinate system ${O}^{w}{x}^{w}{y}^{w}{z}^{w}$ to the camera coordinate system ${O}^{c}{x}^{c}{y}^{c}{z}^{c}$.

For convenience, the camera’s coordinate system is selected as the world coordinate system so that ${{\textit{R}}}^{c}$ is an identity matrix and ${{\bf{t}}}^{c}$ is a zeros vector. By substituting the special ${{\textit{R}}}^{c}$ and ${{\bf{t}}}^{c}$ into Eq. (1), the relationship between $\left({x}^{w},{y}^{w},{z}^{w}\right)$ and $\left({u}^{c},{v}^{c}\right)$ can be simplified as,

2 $${x}^{w}=\frac{{u}^{c}-{u}_{0}^{c}}{{f}_{u}^{c}}{z}^{w}$$

3 $${y}^{w}=\frac{{v}^{c}-{v}_{0}^{c}}{{f}_{v}^{c}}{z}^{w}$$

Similarly, the pinhole model of the projector can be expressed as,

4 $${s}^{p}\left[\begin{array}{c}{u}^{p}\\ {v}^{p}\\ 1\end{array}\right]=\left[\begin{array}{ccc}{f}_{u}^{p} & 0 & {u}_{0}^{p}\\ 0 & {f}_{v}^{p} & {v}_{0}^{p}\\ 0 & 0 & 1\end{array}\right]\left[\begin{array}{ccc}{r}_{11}^{p} & {r}_{12}^{p} & \begin{array}{cc}{r}_{13}^{p} & {t}_{1}^{p}\end{array}\\ {r}_{21}^{p} & {r}_{22}^{p} & \begin{array}{cc}{r}_{23}^{p} & {t}_{2}^{p}\end{array}\\ {r}_{31}^{p} & {r}_{32}^{p} & \begin{array}{cc}{r}_{33}^{p} & {t}_{3}^{p}\end{array}\end{array}\right]\left[\begin{array}{c}{x}^{w}\\ {y}^{w}\\ \begin{array}{c}{z}^{w}\\ 1\end{array}\end{array}\right]$$

where the superscript *p* highlights that we are modeling the projection system; $\left({u}^{p},{v}^{p}\right)$ indicates the coordinate of the “inversed” image of an object point in the pixel coordinate system; ${s}^{p}$ is a scalar; ${f}_{u}^{p}$ and ${f}_{v}^{p}$ (unit: pixel) are the focal lengthes of the projector lens along ${u}^{p}$ and ${v}^{p}$ directions, respectively; $\left({u}_{0}^{p},{v}_{0}^{p}\right)$ is the projector’s principle point coordinate; ${r}_{{ij}}^{p}$ and ${t}_{j}^{p}$ $\left(i=\mathrm{1,2,3}{;j}=\mathrm{1,2,3}\right)$ are the entries of the rotation matrix ${{R}}^{p}$ and translation vector ${{\bf{t}}}^{p}$, respectively. By canceling the unknow scalar ${s}^{p}$, Eq. (4) is re-written as

5 $${u}^{p}=\frac{\left[\left({f}_{u}^{p}\,{r}_{11}^{p}+{r}_{31}^{p}{u}_{0}^{p}\right){x}^{w}+\left({f}_{u}^{p}{r}_{12}^{p}+{r}_{32}^{p}{u}_{0}^{p}\right){y}^{w}+\left({f}_{u}^{p}{r}_{13}^{p}+{r}_{33}^{p}{u}_{0}^{p}\right){z}^{w}+\left({f}_{u}^{p}{t}_{1}^{p}+{t}_{3}^{p}{u}_{0}^{p}\right)\right]}{{r}_{31}^{p}{x}^{w}+{r}_{32}^{p}{y}^{w}+{r}_{33}^{p}{z}^{w}+{t}_{3}^{p}}$$

6 $${v}^{p}=\frac{\left[\left({f}_{v}^{p}{r}_{21}^{p}+{r}_{31}^{p}{v}_{0}^{p}\right){x}^{w}+\left({f}_{v}^{p}{r}_{22}^{p}+{r}_{32}^{p}{v}_{0}^{p}\right){y}^{w}+\left({f}_{v}^{p}{r}_{23}^{p}+{r}_{33}^{p}{v}_{0}^{p}\right){z}^{w}+\left({f}_{v}^{p}{t}_{2}^{p}+{t}_{3}^{p}{v}_{0}^{p}\right)\right]}{{r}_{31}^{p}{x}^{w}+{r}_{32}^{p}{y}^{w}+{r}_{33}^{p}{z}^{w}+{t}_{3}^{p}}$$

FPP calibration is to obtain the values of all the system parameters of the pinhole models given in Eqs. (2), (3), (5) and (6), i.e., $\left({f}_{u}^{c},{f}_{v}^{c},{f}_{u}^{p},{f}_{v}^{p},{r}_{i,j}^{p},{t}_{j}^{p}\right)$. These parameters will be used for the reconstruction of a 3D point $\left({x}^{w},{y}^{w},{z}^{w}\right)$. Thus, given a camera pixel $\left({u}^{c},{v}^{c}\right)$, there are five unknowns $\left({x}^{w},{y}^{w},{z}^{w},{u}^{p},{v}^{p}\right)$, but with only four equations Eqs. (2), (3), (5) and (6). Phase measurement by projecting the sinusoidal fringe patterns has been a widely used choice to precisely determine ${u}^{p}$ and/or ${v}^{p}$, with which, $\left({x}^{w},{y}^{w},{z}^{w}\right)$ can now be easily reconstructed from the above-mentioned equations. As a simple but popularly used example, if ${u}^{p}$ is determined, then $\left({x}^{w},{y}^{w},{z}^{w}\right)$ can be solved from Eqs. (2), (3) and (5). As a result, we have,^17^

7 $${z}^{w}\left({u}^{c},{v}^{c}\right)=\frac{{f}_{u}^{p}{t}_{1}^{p}-\left({u}^{p}-{u}_{0}^{p}\right){t}_{3}^{p}}{\left\{\begin{array}{c}\left[\left({u}^{p}-{u}_{0}^{p}\right){r}_{31}^{p}-{f}_{u}^{p}{r}_{11}^{p}\right]\frac{\left({u}^{c}-{u}_{0}^{c}\right)}{{f}_{u}^{c}}+\\ \left[\left({u}^{p}-{u}_{0}^{p}\right){r}_{32}^{p}-{f}_{u}^{p}{r}_{12}^{p}\right]\frac{\left({v}^{c}-{v}_{0}^{c}\right)}{{f}_{v}^{c}}+\left[\left({u}^{p}-{u}_{0}^{p}\right){r}_{33}^{p}-{f}_{u}^{p}{r}_{13}^{p}\right]\end{array}\right\}}$$

### Phase measurement

For the above-mentioned camera-projector correspondence, i.e., to obtain ${u}^{p}$ and/or ${v}^{p}$ corresponding to $\left({u}^{c},{v}^{c}\right)$, phase measurement through phase-shifting has been widely used in FPP, due to its advantages of high accuracy, resolution, and noise immunity. First, *N*-step phase-shifting patterns with equal phase shifts are generated by a computer according to Eq. (8) and then sent to the projector,

8 $${I}_{n}^{p}\left({u}^{p},{v}^{p}\right)={A}^{p}\left({u}^{p},{v}^{p}\right)+{B}^{p}\left({u}^{p},{v}^{p}\right)\cos \left[\varphi \left({u}^{p},{v}^{p}\right)+\frac{2\pi }{N}\left(n-1\right)\right],\left(n=1,2,\ldots ,N\right)$$

where $A\left({u}^{p},{v}^{p}\right)$ is the background intensity; $B\left({u}^{p},{v}^{p}\right)$ is the fringe amplitude; $\varphi$ is the phase to be solved. The vertical, horizontal, and optimal angle fringes are three typical projected fringes.^17^ Without loss of generality, we take the vertical fringes as an example for analysis and the results can be directly extended to the other two cases. The phase distribution of the vertical fringes is designed as,

9 $$\varphi \left({u}^{p},{v}^{p}\right)=\frac{2\pi }{T}{u}^{p}$$

where *T* is the fringe period.

When the generated fringes are projected onto the test object, the fringes are modulated by the optical properties and the profile of the object surface, i.e., both *A*, *B* will be changed and $\varphi$ re-distributed, where the new phase distribution carries the 3D shape information. The modulated fringe patterns captured by the camera have the same representation as given in Eq. (8), and is given below for clearness,

10 $${I}_{n}^{c}\left({u}^{c},{v}^{c}\right)={A}^{c}\left({u}^{c},{v}^{c}\right)+{B}^{c}\left({u}^{c},{v}^{c}\right)\cos \left[\varphi \left({u}^{c},{v}^{c}\right)+\frac{2\pi }{N}\left(n-1\right)\right],\left(n=1,2,\ldots ,N\right)$$

The phase can then be calculated as,

11 $${\varphi }_{w}\left({u}^{c},{v}^{c}\right)=-{\tan }^{-1}\left\{\frac{\mathop{\sum }\limits_{n=1}^{N}{I}_{n}^{c}\left({u}^{c},{v}^{c}\right)\sin \left[2\pi \left(n-1\right)/N\right]}{\mathop{\sum }\limits_{n=1}^{N}{I}_{n}^{c}\left({u}^{c},{v}^{c}\right)\cos \left[2\pi \left(n-1\right)/N\right]}\right\}$$

where the subscript *w* indicates that the obtained phase is wrapped in a the range from $-\pi$ to $\pi$. In addition, the background intensity and fringe amplitude can also be obtained as,

12 $${A}^{c}\left({u}^{c},{v}^{c}\right)=\frac{\mathop{\sum }\limits_{n=1}^{N}{I}_{n}^{c}}{N}$$

13 $${B}^{c}\left({u}^{c},{v}^{c}\right)=\frac{2}{N}\sqrt{{\left\{\mathop{\sum }\limits_{n=1}^{N}{I}_{n}^{c}\left({u}^{c},{v}^{c}\right)\sin \left[2\pi \left(n-1\right)/N\right]\right\}}^{2}+{\left\{\mathop{\sum }\limits_{n=1}^{N}{I}_{n}^{c}\left({u}^{c},{v}^{c}\right)\cos \left[2\pi \left(n-1\right)/N\right]\right\}}^{2}}$$

From now on, we will focus on analyzing the captured fringe patterns, thus the coordinates $\left({u}^{c},{v}^{c}\right)$ will often be omitted when no confusion is caused.

To obtain the continuous phase distribution, the wrapped phase should be unwrapped as^17^

14 $$\varphi \left({u}^{c},{v}^{c}\right)={\varphi }_{w}\left({u}^{c},{v}^{c}\right)+2\pi k\left({u}^{c},{v}^{c}\right)$$

where $k\left({u}^{c},{v}^{v}\right)$ is the fringe order. When $\left({u}^{c},{v}^{c}\right)$ and $\left({u}^{p},{v}^{p}\right)$ correspond to the same object point, we have,

15 $$\varphi \left({u}^{c},{v}^{c}\right)=\varphi \left({u}^{p},{v}^{p}\right)$$

By substituting Eq. (15) into Eq. (9), we can calculate ${u}^{p}$ as,

16 $${u}^{p}=\frac{\varphi \left({u}^{c},{v}^{c}\right)}{2\pi }T$$

## Two transfer models of noise

According to the principle in Principle of FPP, after calibration, the measurement process of the FPP system can be simply divided into the capturing step and the three computing steps, with six individual models in the model chain, as shown in Fig. 1 and introduced in Introduction. Among all these individual models, we first study the two noise transfer models, **M**_ItoP_ and **M**_PtoG_, because they serve as intermediate bridges for noise transfer. As mentioned earlier, this work focus on precision models, so we pay attention to the variances of ${I}_{n}$, $\varphi$ and ${z}^{w}$.

### M_ItoP_*: from fringes intensity to phase*

According to the phase measurement procedure described in Phase measurement, ${\sigma }_{\varphi }^{2}$ can be quantitatively evaluated. First, we assume that the wrapped phase ${\varphi }_{w}$ can be correctly unwrapping into $\varphi$, which is generally achievable.^41^ Thus, we have

17 $${\sigma }_{\varphi }^{2}\left({u}^{c},{v}^{c}\right)={\sigma }_{{\varphi }_{w}}^{2}\left({u}^{c},{v}^{c}\right)$$

Then, since the wrapped phase is calculated by Eq. (11), we have

18 $${\sigma }_{{\varphi }_{w}}^{2}=\mathop{\sum }\limits_{n=1}^{N}{\left[\frac{\partial {\varphi }_{w}\left({I}_{1}^{c},{I}_{2}^{c},\ldots ,{I}_{N}^{c}\right)}{\partial {I}_{n}^{c}}\right]}^{2}{\sigma }_{{I}_{n}}^{2}$$

where the partial derivative can be derived as

19 $$\frac{\partial {\varphi }_{w}\left({I}_{1}^{c},{I}_{2}^{c},\ldots ,{I}_{N}^{c}\right)}{\partial {I}_{n}^{c}}=-\frac{2}{{B}^{c}N}\sin \left[\varphi +\left(n-1\right)\frac{2\pi }{N}\right]$$

Finally, by substituting Eqs. (18) and (19) into Eq. (17), the ItoP precision conversion can be expressed as,

20 $${{\bf{M}}}_{{\rm{ItoP}}}\!\!:{\sigma }_{\varphi }^{2}={\left(\frac{2}{{B}^{c}N}\right)}^{2}\mathop{\sum }\limits_{n=1}^{N}{\sin }^{2}\left[\varphi +\left(n-1\right)\frac{2\pi }{N}\right]{\sigma }_{{I}_{n}}^{2}$$

The model’s name is put in the equation for easy reference, which will be done for all the models to be introduced.

### M_PtoG_*: from phase to geometry*

Equation (7) reconstructs ${z}^{w}$ with three groups of variables/parameters: (i) a camera pixel $\left({u}^{c},{v}^{c}\right)$ is specified for 3D reconstruction; (ii) given the specified $\left({u}^{c},{v}^{c}\right)$, ${u}^{p}$ is calculated by using Eq. (16) whose precision is affected by the phase measurement result; (iii) all the other parameters appearing in the right hand of Eq. (7) are system-related, pre-calibrated, and assumed to be perfectly known. Thus, ${u}^{p}$ is the only variable for the consideration of the measurement precision so that we have,

21 $${\sigma }_{z}^{2}={\left(\frac{\partial {z}^{w}}{\partial {u}^{p}}\right)}^{2}{\sigma }_{{u}^{p}}^{2}$$

Firstly, we obtain $\partial {z}^{w}/\partial {u}^{p}$ from Eq. (7) as,

22 $${\left(\frac{\partial {z}^{w}}{\partial {u}^{p}}\right)}_{F}=\frac{{\left[{r}_{31}^{p}\frac{\left({u}^{c}-{u}_{0}^{c}\right)}{{f}_{u}^{c}}{z}^{w}+{r}_{32}^{p}\frac{\left({v}^{c}-{v}_{0}^{c}\right)}{{f}_{v}^{c}}{z}^{w}+{r}_{33}^{p}{z}^{w}+{t}_{3}^{p}\right]}^{2}}{{f}_{u}^{p}\left[\left({r}_{11}^{p}{t}_{3}^{p}-{r}_{31}^{p}{t}_{1}^{p}\right)\frac{\left({u}^{c}-{u}_{0}^{c}\right)}{{f}_{u}^{c}}+\left({r}_{12}^{p}{t}_{3}^{p}-{r}_{32}^{p}{t}_{1}^{p}\right)\frac{\left({v}^{c}-{v}_{0}^{c}\right)}{{f}_{v}^{c}}+\left({r}_{13}^{p}{t}_{3}^{p}-{r}_{33}^{p}{t}_{1}^{p}\right)\right]}$$

where *F* indicates that it is a full model. We mention that the direct derivation of Eq. (22) from Eq. (7) is lengthy, and an easier shortcut is to refer to the derivation of $\partial {u}^{p}/\partial {z}^{w}$ in Ref. ^17^. To estimate the variance of ${z}^{w}$ according to Eq. (21), we yet need to know the variance of ${u}^{p}$, which can be calculated from Eq. (16) as,

23 $${\sigma }_{{u}^{p}}^{2}={\left(\frac{T}{2\pi }\right)}^{2}{\sigma }_{\varphi }^{2}$$

Substituting Eqs. (22) and (23) into Eq. (21), **M**_PtoG_ precision transfer model under ideal calibration can be obtained as,

24 $${{\bf{M}}}_{{\rm{PtoG}}-{\rm{F}}}\!\!:{\sigma }_{z}^{2}={\left(\frac{T}{2\pi }\right)}^{2}{\left\{\frac{{\left[{r}_{31}^{p}\frac{\left({u}^{c}-{u}_{0}^{c}\right)}{{f}_{u}^{c}}{z}^{w}+{r}_{32}^{p}\frac{\left({v}^{c}-{v}_{0}^{c}\right)}{{f}_{v}^{c}}{z}^{w}+{r}_{33}^{p}{z}^{w}+{t}_{3}^{p}\right]}^{2}}{{f}_{u}^{p}\left[\left({r}_{11}^{p}{t}_{3}^{p}-{r}_{31}^{p}{t}_{1}^{p}\right)\frac{\left({u}^{c}-{u}_{0}^{c}\right)}{{f}_{u}^{c}}+\left({r}_{12}^{p}{t}_{3}^{p}-{r}_{32}^{p}{t}_{1}^{p}\right)\frac{\left({v}^{c}-{v}_{0}^{c}\right)}{{f}_{v}^{c}}+\left({r}_{13}^{p}{t}_{3}^{p}-{r}_{33}^{p}{t}_{1}^{p}\right)\right]}\right\}}^{2}{\sigma }_{\varphi }^{2}$$

This model is applicable for any practical systems.

Next, it is seen from Eq. (22) that $\partial {u}^{p}/\partial {z}^{w}$ is not constant but varies with $\left({u}^{c},{v}^{c}\right)$. This implies that an FPP system has inherent spatially non-uniform error distribution. Fortunately, for a typical FPP system with a camera and projector placed on left and right, ${r}_{11}^{p}$, ${r}_{22}^{p}$ and ${r}_{33}^{p}$ are closed to 1, and the other entries of ${{\textit{R}}}^{p}$ are much small. Additionally, the camera’s focal lengths (unit: pixel) are larger than the detector’s pixel number, and thus $\left({u}^{c}-{u}_{0}^{c}\right)/{f}_{u}^{c}$, $\left({v}^{c}-{v}_{0}^{c}\right)/{f}_{v}^{c}$ are less than 1. Based on the above two facts, in both the numerator and the denominator of Eq. (22), the first two terms in the bracket can be omitted compared with the third term. Equation (22) can then be approximated into a simpler form,

25 $${\left(\frac{\partial {z}^{w}}{\partial {u}^{p}}\right)}_{A}\approx \frac{{\left({r}_{33}^{p}{z}^{w}+{t}_{3}^{p}\right)}^{2}}{{f}_{u}^{p}\left({r}_{13}^{p}{t}_{3}^{p}-{r}_{33}^{p}{t}_{1}^{p}\right)}$$

Accordingly, we have the following approximated model,

26 $${{\bf{M}}}_{{\rm{PtoG}}-{\rm{A}}}\!\!:{\sigma }_{z}^{2}={\left(\frac{T}{2\pi }\right)}^{2}{\left[\frac{{\left({r}_{33}^{p}{z}^{w}+{t}_{3}^{p}\right)}^{2}}{{f}_{u}^{p}\left({r}_{13}^{p}{t}_{3}^{p}-{t}_{1}^{p}{r}_{33}^{p}\right)}\right]}^{2}{\sigma }_{\varphi }^{2}$$

where A indicates the approximation. This model is more concise and practical both for guiding FPP system design and for precision analysis of an already established FPP system. However, caution must be taken on whether the approximation is valid, for which, we suggest to numerically evaluate the following relative error between two partial derivatives when the system parameters are available,

27 $${E}_{r}=\left|\,\frac{{\left(\frac{\partial {z}^{w}}{\partial {u}^{p}}\right)}_{A}-{\left(\frac{\partial {z}^{w}}{\partial {u}^{p}}\right)}_{F}}{{\left(\frac{\partial {z}^{w}}{\partial {u}^{p}}\right)}_{F}}\right|\times 100 \%$$

${E}_{r}$ is determined by the intrinsic and extrinsic parameters of FPP, $\left({u}^{c},{v}^{c}\right)$, and the measured depth ${z}^{w}$. We define the maximum relative error, ${E}_{{r}_{\max }}$ as the maximum value of ${E}_{r}$ at a depth ${z}^{w}$. As an example, for the verification in Validation of the theoretical reconstruction precision of FPP, we set up an FPP system and calibrate it within a depth range from 850 mm to 1000 mm. With the calibration parameters and the depth value, we are able to calculate ${E}_{{r}_{\max }}$ using Eq. (27) ranging from 1.436% to 1.441% across this depth range. We then modify the configuration of the system in a less favorable manner and found that ${E}_{{r}_{\max }}$ do increase to $\left[8.748 \% ,8.881 \% \right]$ across the calibration depth range from 380 mm to 480 mm. Details of these two systems are given in Validation of the theoretical reconstruction precision of FPP. In addition, we evaluate the system recommended by Zhang in his book as it is “capable of achieving good sensitivity and compact design” based on his experience,^13^ and calculate its ${E}_{{rmax}}$ to be 1.117% at the designed measuring distance. Thus **M**_PtoG-A_ can be used when ${E}_{{r}_{\max }}$ is small (say, less than 5%), while **M**_PtoG-F_ should be used otherwise.

Finally, according to our analysis,^17^ for the vertical fringe projection, the optimal precision can be achieved when the epipolar line of the camera and projector is horizontal. In this case, the rotation matrix is an identity matrix and ${t}_{3}^{p}$ = 0, which gives

28 $${{\bf{M}}}_{{\rm{PtoG}}-{\rm{E}}}\!\!:{\sigma }_{z}^{2}={\left(\frac{T}{2\pi }\right)}^{2}{\left[\frac{{\left({z}^{w}\right)}^{2}}{{f}_{u}^{p}{t}_{1}^{p}}\right]}^{2}{\sigma }_{\varphi }^{2}$$

where E stands for epipolar. This model is useful for precision prediction, and also clearly shows the possibly ways of precision improvement: to reduce the fringe period *T*, the object distance ${z}^{w}$ and noise level ${\sigma }_{\varphi }$, and to increase the focal length ${f}_{u}^{p}$ and the baseline ${t}_{1}^{p}$ between the camera and projector. Among them, ${\sigma }_{\varphi }$ will be elaborated in **M**_P_: model the phase noise using the new **M**_I-C_; *T* will be discussed in The optimal period of the projected fringe patterns; others are often constrained by a practical measurement task. We mention that when the epipolar line is not horizontal, using the optimal fringe angle for projection^17^ can also achieve the same precision.

## Four stage models of noise

In this section, we introduce and explain the four stage models, **M**_C_, **M**_I_, **M**_P_ and **M**_G_, in Sections **M**_C_: an existing camera noise model to be adopted to **M**_G_: integrated noise model for the reconstructed geometry, respectively.

### M_C_: *an existing camera noise model to be adopted*

**Fig. 2:**
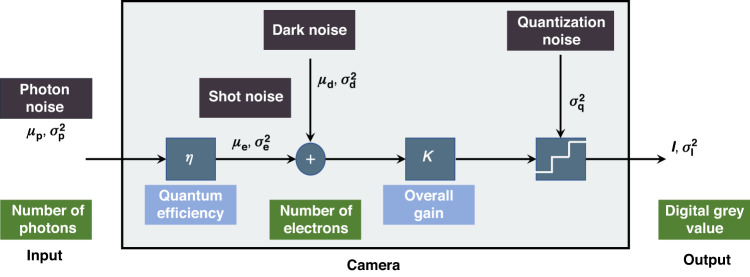
Mathematical model of a camera, adapted from^33^ with minor modification

We first explain an existing noise model which we will adopt into FPP. The comparison with the tradition Gaussian model will be given in Comparison with the tradition Gaussian model. Figure 2 shows the mathematical model of a linear camera sensor which converts photons hitting a pixel area during the exposure time into a digital number (DN) which is a gray value of the corresponding pixel in the captured image. We follow the convention in the European Machine Vision Association (EMVA) Standard 1288,^33^ and use DN as the unit of the gray value. During the exposure time, the process of converting the average number of photons $\left({\mu }_{p}\right)$ into the average number of electrons $\left({{\mu }_{e},{\rm{unit}}\!\!:{e}}^{-}\right)$ can be determined by the quantum efficiency $\eta$ of the sensor,

29 $${\mu }_{e}=\eta {\mu }_{p}$$

Since the number of electrons fluctuates statistically with Poisson probability distribution,^33^ we have,

30 $${\sigma }_{e}^{2}={\mu }_{e}$$

In addition, a sensor also generates electrons ${\mu }_{d}$ due to the influence of temperature without light. Both types of the electrons are converted into a voltage, amplified, and finally converted into a digital signal *I* (unit:DN) by an analog digital converter (ADC). The whole process can be described as,

31 $$I=K\left({\mu }_{e}+{\mu }_{d}\right)=K{\mu }_{e}+{I}_{{dark}}$$

where $K({\rm{unit}}\!\!:{\rm{DN}}{\left({{\rm{e}}}^{-}\right)}^{-1})$ is the overall gain of a camera; ${I}_{{dark}}=K{\mu }_{d}$ is called dark signal. Because all noise sources are independent, the total variance of the digital signal $I$ can be expressed as,

32 $${\sigma }_{I}^{2}={{K}^{2}{\sigma }_{e}^{2}+K}^{2}{\sigma }_{d}^{2}+{\sigma }_{q}^{2}$$

which includes three noise sources: (i) shot noise with a variance of ${\sigma }_{e}^{2}$ $\left({{\rm{unit}}\!\!:\left({e}^{-}\right)}^{2}\right)$; (ii) all the noise sources related to the sensor read out and amplifier circuits represented by ${\sigma }_{d}^{2}$ $\left({{\rm{unit}}\!\!:\left({e}^{-}\right)}^{2}\right)$; (iii) the quantization noise with a variance of ${\sigma }_{q}^{2}=1/12\left({{\rm{unit}}\!\!:\left({\rm{DN}}\right)}^{2}\right)$.^42^

Substituting Eqs. (30) and (31) into Eq. (32) gives,

33 $${\sigma }_{I}^{2}={\sigma }_{{dark}}^{2}+K\left(I-{I}_{{dark}}\right)$$

34 $${\sigma }_{{dark}}^{2}{=K}^{2}{\sigma }_{d}^{2}+{\sigma }_{q}^{2}$$

Equation (33) is central to the characterization of the sensor for camera manufacturers to test the specifications of a camera, and is known as the photon transfer curve (PTC) in EMVA 1288.^33^ For users, it is more convenient to re-write this equation as,

35 $${{\bf{M}}}_{{\rm{C}}}\!\!:{\sigma }_{I}^{2}={C}_{n}+{KI}$$

36 $${C}_{n}={\sigma }_{{dark}}^{2}-K{I}_{{dark}}$$

where **M**_C_ is used to denote the camera model. It is clear that the variance of intensity is proportional to the image intensity, which is seldom noticed in the FPP literature.

### M_I_: *model the fringe intensity noise*

Applying **M**_C_ to the fringe intensities is straightforward but significant, as it will influence the entire model chain. By submitting the acquired phase-shifting fringe patterns introduced in Phase measurement into Eq. (35), we have

37 $${{\bf{M}}}_{{\rm{I}}-{\rm{C}}}\!\!:{\sigma }_{{I}_{n}}^{2}={C}_{n}+K\left\{{A}^{c}+{B}^{c}\cos \left[\varphi +\left(n-1\right)\frac{2\pi }{N}\right]\right\},n=1,2,3\ldots N$$

where C indicates that this model is based on the new camera model.

### M_P_: *model the phase noise using the new* M_I-C_

Now, based on **M**_I-C_, we develop three new phase models for different purposes. By substituting **M**_I-C_ in Eq. (37) into **M**_ItoP-F_ in Eq. (20), the phase variance is obtained as,

38 $${{\bf{M}}}_{{\rm{P}}-{\rm{F}}}\!\!:{\sigma }_{\varphi }^{2}=\frac{2}{{\left({B}^{c}\right)}^{2}N}\left({C}_{n}+K{A}^{c}\right)$$

where F stands for a full phase noise model. This equation clearly indicates that the variance of the phase is determined by the parameters (${A}^{c}$ and ${B}^{c}$) of the fringes and the parameters (*K* and ${C}_{n}$) of a camera, where *K* and ${C}_{n}$ can be obtained from the manufacture of the sensor, e.g. FLIR recently released 2022 Camera Sensor Review testing using EMVA 1288.^43^ Once the acquired fringe intensity is set, the camera parameters fully determine the variance of the phase noise.

According to the *K* value provided by FLIR,^43^ the term of ${C}_{n}$ can be negligible compared with *K*${A}^{c}$ in actual measurement, especially when a camera captures images with 10 bits greyscale value or higher. For example, for the camera used in this study (8 bits), $K{A}^{c}/{C}_{n} > 10$, which will be seen in Validation of the camera noise model. Thus, Eq. (38) can be approximated as,

39 $${{\bf{M}}}_{{\rm{P}}-{\rm{A}}}\!\!:{\sigma }_{\varphi }^{2}\approx \frac{2}{{\left({B}^{c}\right)}^{2}N}K{A}^{c}$$

where A in the subscript stands for approximation. This model can be used for quick estimation. However, **M**_P-A_ still relies on the background intensity ${A}^{c}$ and fringe amplitude ${B}^{c}$, which will change for different object surface properties.

In a special case where the acquired fringe image is close to the saturation value, the overall gain of a camera can be expressed as,^33^

40 $$K\approx \frac{{I}_{{Sat}}}{{\mu }_{e.{Sat}}}$$

where ${\mu }_{e.{sat}}$ $\left({\rm{unit}}\!\!:{e}^{-}\right)$ is the saturation capacity. For a fringe pattern expressed in Eq. (10), the maximum intensity is ${I}_{{Sat}}={A}^{c}+{B}^{c}$. Further consider the case where ${A}^{c}\approx {B}^{c}$, we have ${I}_{{Sat}}\approx 2{B}^{c}$, and then Eq. (40) can be re-written as,

41 $$K\approx \frac{2{B}^{c}}{{\mu }_{e.{Sat}}}$$

Substituting Eq. (41) into Eq. (39), the phase noise can be simplified as,

42 $${{\bf{M}}}_{{\rm{P}}-{\rm{S}}}\!\!:{\sigma }_{\varphi }^{2}\approx \frac{4}{N{\mu }_{e.{Sat}}}$$

which is named **M**_P-S_ where S stands for saturation. The interesting factors of this model are that, first, the noise variance is no longer dependent on either ${A}^{c}$ or ${B}^{c}$, allowing us to clearly know the measurement precision limit for an ideal FPP system, and second, ${\mu }_{e.{Sat}}$ is also a basic parameter provided by the camera manufacturer and thus very convenient to use. This model is most useful to theoretically guide the selection of a sensor.

Table 1 shows the overall gain and saturation capacity of several commonly used sensors, obtained from 2022 Camera Sensor Review-Mono released by FILR company.^43^ Note that the gain provided by FLIR is the number of electrons required to increase the pixel value from a 16-bit greyscale value to one DN higher.^43^ Therefore, if 8-bit, 10-bit and 12-bit greyscale values are used, users need to be divided these parameters in Table 1 by ${2}^{8}$, ${2}^{6}$ and ${2}^{4}$, respectively. (Note that ${C}_{n}$ cannot been obtained by using a similar conversion way. If you require a ${C}_{n}$ for a specific bit number, it is advisable to contact the camera manufacturer).

**Table 1:** the overall gain and saturation capacity of the typical sensors

Model Number	Sensor	Gain ${\boldsymbol{K}}\left({\mathbf{DN}}{\left({{\mathbf{e}}}^{\boldsymbol{-}}\right)}^{\boldsymbol{-1}}\right)$	Saturation Capacity ${\boldsymbol{{\mu }_{e.{Sat}}}}$ $\left({\boldsymbol{e}}^{\boldsymbol{-}}\right)$
BFS-U3-51S5M-C	Sony: IMX250	5.7	10,970
GS3-U3-23S6M-C	Sony: IMX174	1.92	33,022
GS3-U3-41C6M-C	COMSIS: CMV4000	6.27	9,983
BFLY-PGE-23S6M-C	Sony: IMX249	1.92	33,022
BFS-U3-16S7M-C(LCG)	Sony: IMX432	0.65	98,965.6

### M_G_: *integrated noise model for the reconstructed geometry*

By integrating an **M**_P_ model (**M**_P-F_, **M**_P-A_ or **M**_P-S_) developed in **M**_P_: model the phase noise using the new **M**_I-C_ and a **M**_PtoG_ model (**M**_PtoG-F_, **M**_PtoG-A_ or **M**_PtoG-E_) developed in **M**_PtoG_: from phase to geometry, we can obtain nine different combinations and thus nine different versions of **M**_G_, among which the following four models are most useful and significant. The first version integrates **M**_P-F_ (full model) and **M**_PtoG-F_ (full model), which accurately evaluate of the measurement precision of a constructed FPP system under any circumstances, but is also most complicated:

43 $${{\rm{M}}}_{{\rm{G}}-{\rm{F}}}\!\!:{\sigma }_{z}^{2}=\frac{2}{{\left({B}^{c}\right)}^{2}N}{\left(\frac{T}{2\pi }\right)}^{2}{\left\{\frac{{\left[{r}_{31}^{p}\frac{\left({u}^{c}-{u}_{0}^{c}\right)}{{f}_{u}^{c}}{z}^{w}+{r}_{32}^{p}\frac{\left({v}^{c}-{v}_{0}^{c}\right)}{{f}_{v}^{c}}{z}^{w}+{r}_{33}^{p}{z}^{w}+{t}_{3}^{p}\right]}^{2}}{{f}_{u}^{p}\left[\left({r}_{11}^{p}{t}_{3}^{p}-{r}_{31}^{p}{t}_{1}^{p}\right)\frac{\left({u}^{c}-{u}_{0}^{c}\right)}{{f}_{u}^{c}}+\left({r}_{12}^{p}{t}_{3}^{p}-{r}_{32}^{p}{t}_{1}^{p}\right)\frac{\left({v}^{c}-{v}_{0}^{c}\right)}{{f}_{v}^{c}}+\left({r}_{13}^{p}{t}_{3}^{p}-{r}_{33}^{p}{t}_{1}^{p}\right)\right]}\right\}}^{2}\left(K{A}^{c}+{C}_{n}\right)$$

The second version integrates **M**_P-F_ (full model) and **M**_PtoG-A_ (approximation mode) when ${C}_{n}$ is available from the camera manufacturer:

44 $${{\bf{M}}}_{{\rm{G}}-{\rm{A}}1}\!\!:{\sigma }_{z}^{2}={\frac{2}{{\left({B}^{c}\right)}^{2}N}\left(\frac{T}{2\pi }\right)}^{2}{\left[\frac{{\left({r}_{33}^{p}{z}^{w}+{t}_{3}^{p}\right)}^{2}}{{f}_{u}^{p}\left({r}_{13}^{p}{t}_{3}^{p}-{t}_{1}^{p}{r}_{33}^{p}\right)}\right]}^{2}\left(K{A}^{c}+{C}_{n}\right)$$

The third version integrates **M**_P-A_ (approximation model) and **M**_PtoG-A_ (approximation model) when ${C}_{n}$ is not available from the camera manufacturer:

45 $${{\bf{M}}}_{{\rm{G}}-{\rm{A}}2}\!\!:{\sigma }_{z}^{2}={\frac{2}{{\left({B}^{c}\right)}^{2}N}\left(\frac{T}{2\pi }\right)}^{2}{\left[\frac{{\left({r}_{33}^{p}{z}^{w}+{t}_{3}^{p}\right)}^{2}}{{f}_{u}^{p}\left({r}_{13}^{p}{t}_{3}^{p}-{t}_{1}^{p}{r}_{33}^{p}\right)}\right]}^{2}K{A}^{c}$$

The fourth version integrates **M**_P-S_ and **M**_PtoG-E_ which is simplest and can be used to predict the theoretical precision limit before system development because the fringe intensity approaches saturation in **M**_P-S_ and the fringe angel is optimal in **M**_PtoG-E_:

46 $${{\bf{M}}}_{{\rm{G}}-{\rm{L}}}\!\!:{\sigma }_{z}^{2}={\left(\frac{T}{2\pi }\right)}^{2}{\left[\frac{{\left({z}^{w}\right)}^{2}}{{f}_{u}^{p}{t}_{1}^{p}}\right]}^{2}\frac{4}{N{\mu }_{e.{Sat}}}$$

### Comparison with the tradition Gaussian model

The Gaussian noise model has been used in FPP almost exhaustively. It assumes that the noise at a pixel is Gaussian with a mean of zero and a variance of ${\sigma }_{I}^{2}$ which is shared by all the pixels in all the phase-shifted fringe patterns.^25–27,31^ Consequently, the variance of the fringe intensity can be written as

47 $${{\bf{M}}}_{{\rm{I}}-{\rm{G}}}\!\!:{\sigma }_{{I}_{n}}^{2}={\sigma }_{I}^{2},n=1,2,3\ldots N$$

Combining **M**_I-G_ with **M**_ItoP_ model gives the following phase variance,

48 $${{\bf{M}}}_{{\rm{P}}-{\rm{G}}}:{\sigma }_{\varphi }^{2}=\frac{2}{{\left({B}^{c}\right)}^{2}N}{\sigma }_{I}^{2}$$

Further combing **M**_P-G_ with **M**_PtoG-F_ gives

49 $${{\bf{M}}}_{{\rm{G}}-{\rm{G}}}\!\!:{\sigma }_{z}^{2}={\frac{2}{{\left({B}^{c}\right)}^{2}N}\left(\frac{T}{2\pi }\right)}^{2}{\left\{\frac{{\left[{r}_{31}^{p}\frac{\left({u}^{c}-{u}_{0}^{c}\right)}{{f}_{u}^{c}}{z}^{w}+{r}_{32}^{p}\frac{\left({v}^{c}-{v}_{0}^{c}\right)}{{f}_{v}^{c}}{z}^{w}+{r}_{33}^{p}{z}^{w}+{t}_{3}^{p}\right]}^{2}}{{f}_{u}^{p}\left[\left({r}_{11}^{p}{t}_{3}^{p}-{r}_{31}^{p}{t}_{1}^{p}\right)\frac{\left({u}^{c}-{u}_{0}^{c}\right)}{{f}_{u}^{c}}+\left({r}_{12}^{p}{t}_{3}^{p}-{r}_{32}^{p}{t}_{1}^{p}\right)\frac{\left({v}^{c}-{v}_{0}^{c}\right)}{{f}_{v}^{c}}+\left({r}_{13}^{p}{t}_{3}^{p}-{r}_{33}^{p}{t}_{1}^{p}\right)\right]}\right\}}^{2}{\sigma }_{I}^{2}$$

We now compare our adopted camera model **M**_C_ with the Gaussian model **M**_G_. Referring to Eq. (35) and also noting that ${C}_{n}$ is ignorable, we have ${\sigma }_{I}^{2}={KI}$ which provides a large range of variance. For example, when *I* changes from 30 DN to 240 DN when different phase shifts are applied, the variance will change from 30 *K* to 240 *K*, i.e., the variance can be different by 8 times. However, in the Gaussian model, the variance is assumed to be constant. This will affect the subsequent evaluation of the precision at all the stages in the model chain. In addition, in the Gaussian noise model, the noise variance ${\sigma }_{I}^{2}$ is unknown in advance and has to be measured, and the measurement result varies with the measured object and the surrounding environment, which is undesirable.

For easy reference, Table 2 summarizes the most useful phase and geometry models.

**Table 2:** Summary of the phase and geometry models for easy reference

Camera noise models	Phase noise models		Important geometric models		
	${\boldsymbol{\sigma }}_{\boldsymbol{\varphi }}^{\boldsymbol{2}}$		${\boldsymbol{\sigma }}_{\boldsymbol{z}}^{\boldsymbol{2}}$		
**M**_I-G_ **(Gaussian)**	**M**_P-G_: Eq. (48)	Gaussian model	**M**_G-G_: Eq. (49)	Full model	The Gaussian assumption
**M**_I-C_ **(Non-Gaussian)**	**M**_P-F_: Eq. (38)	Full phase model	**M**_G-F_: Eq. (43)	Full model	Generally applicable
			**M**_G-A1_: Eq. (44)	The first approximation model	When ${C}_{n}$ is provided
	**M**_P-A_: Eq. (39)	Approximation model	**M**_G-A2_: Eq. (45)	The second approximation model	When ${C}_{n}$ is not provided
	**M**_P-S_: Eq. (42)	Saturation model	**M**_G-L_: Eq. (46)	Limit model	To predict the theoretical precision limit

## Experimental verification

In this section, we carefully verify the following key aspects to support our theoretical analysis:

(i) Since this is the first time to adopt the camera noise model for FPP, we verify this model by our measurement in detail (Validation of the camera noise model);
(ii) We have stated in **M**_C_: an existing camera noise model to be adopted that the camera noise is dominant comparing with other error source. We will measure the influence of the projector to show that it is indeed ignorable (Validation of the projector noise);
(iii) We further verify the above statement on all error sources, by showing that the measured phase variance influenced by all error sources in a real experiment agrees perfectly with that predicted by our proposed phase models with only camera noise (Validation of the phase noise models);
(iv) We finally verify the reconstruction precision which is one of the most important indicators of FPP (Validation of the theoretical reconstruction precision of FPP).

In this study, all experiments are conducted using the same camera (Model: MER2-502-79U3M-L, resolution: $2048\times 2448$, sensor: Sony IMX250).

### Validation of the camera noise model

**Fig. 3:**
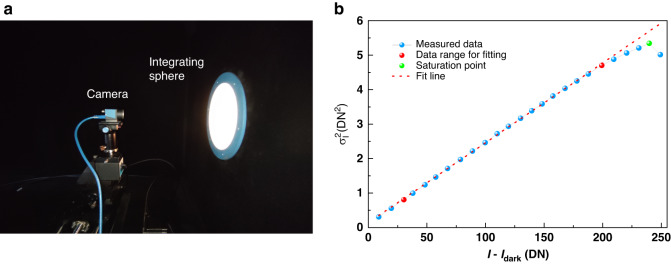
Testing the PTC of the camera. **a** Live testing of the camera; **b** PTC of the camera

In this section, we validate the camera noise model expressed in Eq. (35). We measure the PTC of the camera using an integrating sphere which is able to illuminate the image sensor homogeneously.^44^ Figure 3a shows the setup in a dark room with an illuminance of lower than ${10}^{-4}$ lx. During the test, the exposure time of the camera is set to 20 ms and images are captured as 8-bit monochromatic and saved in a raw format. The brightness of the integrating sphere increases gradually from the initial dark field, so that the mean image gray level increases from its initial small value to 250 DN. The increment interval is approximately 10 DN. In the dark field, we measure the mean value of the gray value of all the pixels as

50 $${I}_{{dark}}=0.9987{\rm{D}}{\rm{N}}$$

From each captured image, the mean value of the gray value of all the pixels is calculated as $I$, while the variance of all these pixels is used as ${\sigma }_{I}^{2}$. We then plot ${\sigma }_{I}^{2}$ with respect to $I-{I}_{{dark}}$ as blue dots in Fig. 3b, which serves as our measured PTC curve.

#### Validation of the camera’s linear noise model

We take the data between the two red points (including the red points) in Fig. 3b and fit them as a line by least squares fitting. The fit line is also shown in Fig. 3b as a red dotted line.

51 $${\sigma }_{I}^{2}=0.1419+0.0232\left(I-{I}_{{dark}}\right)$$

The coefficient of determination ${R}^{2}$^45^ is calculated $0.9996$, demonstrating perfect linearity between ${\sigma }_{I}^{2}$ and $I-{I}_{{dark}}$. This measurement result clearly shows that the noise variance indeed changes according to the input intensity. Such a phenomenon should not be ignored in precision evaluation.

#### Validation of the camera’s key parameters

According to Eq. (51), the estimated slope gives the overall gain *K* = 0.0232 ${\rm{DN}}{\left({{\rm{e}}}^{-}\right)}^{-1}$. The saturation gray value is *I*_*Sat*_ = 240 DN, which is shown as the green point in Fig. 3b. Therefore, the saturation capacity can be obtained as,

52 $${\mu }_{e.{Sat}}=\frac{{I}_{{Sat}}}{K}=10345{e}^{-}$$

We compare our results with other two independent testing results:

(i) The FILR’s result has been provided in the first row of Table 1 where their $K$ value is measured for 16-bit greyscale. We adapt the result to 8-bit by converting the *K* value as 5.7/2^8^ = 0.0222 and adapt ${\mu }_{e.{Sat}}$ accordingly. The result in shown in the first row of Table 3.

**Table 3:** comparison of the camera’s key parameters

	*Gain* ${\boldsymbol{K}}({\mathbf{DN}}{\left({{\mathbf{e}}}^{\boldsymbol{-}}\right)}^{\boldsymbol{-1}})$	*Saturation Capacity* ${\boldsymbol{\mu}} _{\boldsymbol{e.{Sat}}}$ $\left({{\mathbf{e}}}^{\boldsymbol{-}}\right)$	Relative difference of *K*$\,\left( {\boldsymbol{\%}} \right)$	Relative difference of ${\boldsymbol{\mu}} _{\boldsymbol{{e.{Sat}}}}$ $\left({\boldsymbol{ \%}} \right)$
FILR’s results	0.0222	10,970	-	-
DaHeng’s results	0.0237	10,325	6.8	5.9
Our results	0.0232	10,345	4.5	5.7
(ii) The data shared by DaHeng company^46^ undergo a similar conversion from 10-bit to 8-bit.
(iii) The relative differences are computed by using the FILR’s results as the ground truth. The differences are less than 7% and considered as consistent.

#### Validation of the significance of ***C***_***n***_

Substituting Eq. (50) into Eq. (51) gives,

53 $${\sigma }_{I}^{2}=0.1187+0.0232I$$

So, the value of ${C}_{n}$ is $0.1187{\left({\rm{DN}}\right)}^{2}$. According to Eq. (38), we can calculate the ratio for the following two particular cases:

54 $$\frac{K{A}^{c}}{{C}_{n}}=\left\{\begin{array}{c}10,{when}\,{A}^{c}=52\\ 19,{when}\,{A}^{c}=100\end{array}\right.$$

Thus, ${C}_{n}$ is smaller by more than an order of magnitude and ignorable for theoretical consideration, to support our statement in **M**_I_: model the fringe intensity noise. To be safer, since all the above-mentioned camera parameters are available from the camera manufacturer, users are encouraged to evaluate the above ratio in actual measurement.

Since our experiment well validates the camera noise model, our measured parameters in Table 3 will be used in all our later verifications.

### Validation of the projector noise

**Fig. 4:**
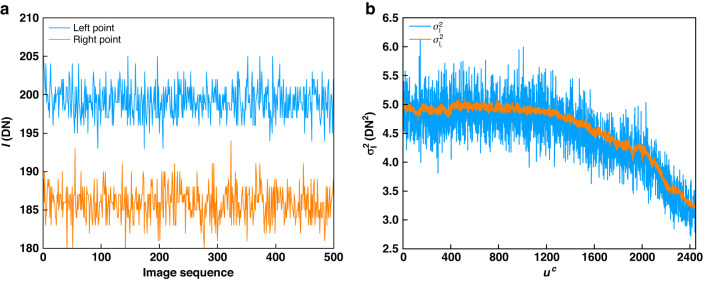
Validation of projector noise. **a** The temporal evolution of intensity at randomly pixels; **b** the distribution of$\,{\sigma }_{I}^{2}$ and ${\sigma }_{{I}_{r}}^{2}$ along the middle row of pixels on the camera

In this experiment, we study the influence of the projector’s stability on the captured image intensity. The projector used is a DLP projector (Model: DLP4500, resolution: 1140×912) and the camera is equipped with a VTG-1614-M4 lens with a focal length of 16 mm. Before starting to verify, the projector was turned on for thirty minutes for thermal equilibrium, where the LED of the projector is set to green light. Then, the projector projects, repeatedly for 500 times, an image with a uniform grayscale onto a white plate. The camera captures 500 images. To check the stability of the projector, the temporal evolution of two randomly selected pixels, one from the left of the camera with a coordinate of (592, 800) and the other from right with a coordinate of (1536, 1836), are shown in Fig. 4a. We interestingly find that these two pixels show different light intensity (the left pixel has higher intensity than the right pixel). This is a common phenomenon mainly due to the non-uniformity and directional scattering of the plate, and the angle between the camera’s viewing angle and the direction of the incident light,^47^ and the illumination of the projector are not uniform, with a typical uniformity of around 90%.^48^

We now statistically examine the projector’s stability. We calculate the mean and variance for each pixel separately as,

55 $$\bar{I}\left({u}^{c},{v}^{c}\right)=\frac{1}{L}\mathop{\sum }\limits_{l=1}^{L}{I}_{l}\left({u}^{c},{v}^{c}\right)$$

56 $${\sigma }_{I\left({u}^{c},{v}^{c}\right)}^{2}=\frac{1}{L}\mathop{\sum }\limits_{l=1}^{L}{\left[{I}_{l}\left({u}^{c},{v}^{c}\right)-\bar{I}\left({u}^{c},{v}^{c}\right)\right]}^{2}$$

where ${I}_{l}\left({u}^{c},{v}^{c}\right)$ is the intensity of the ${l}^{{th}}$ captured image at the pixel $\left({u}^{c},{v}^{c}\right)$ and the total frame number is *L* = 500 in our test.

We then use the mean intensity $\bar{I}\left({u}^{c},{v}^{c}\right)$ to predict the intensity variance from Fig. 3b as a reference (${\sigma }_{{I}_{r}}^{2}$) which is solely due to the camera’s influence. Linear interpolation is used when the input intensity falls between two available data in Fig. 3b. For a qualitative observation, Fig. 4b shows the distribution of$\,{\sigma }_{I}^{2}$ and ${\sigma }_{{I}_{r}}^{2}$ along the middle row of pixels on the camera. The difference between the measured variance and the reference variance at each pixel is very small, and the latter appears to be a smoothed version of the former. We also quantitatively calculate the mean and variance of the differences of all the pixels to be $-0.0864{\left({\rm{DN}}\right)}^{2}$ and $0.0837{\left({\rm{DN}}\right)}^{4}$, respectively, which convincingly demonstrates that the measured variance, including the influence of the projector’s stability (and implicitly, all other possible disturbances), perfectly matches that without considering such influence. Thus, when evaluating a system’s precision limit, we can safely ignore the projector’s influence. We mention that the measured data should be larger than the theoretical predication, whereas in our case, the former is a little larger, which is attributed to the imperfection in the experimental data in both Figs. 3b and 4.

### Validation of the phase noise models

In this section, we validate the proposed phase noise models with the following procedure:

(i) Sequentially project 500 sets of fringe patterns with ${A}^{p}={B}^{p}=127.5$. Each set includes 9-step phase-shifted fringe patterns with a period of 21 pixels. Capture all 500 sets of fringe patterns. Define the regional of interest (ROI) for validation;
(ii) Calculate the wrapped phase ${\varphi }_{{wl}}$, background intensity ${A}_{l}^{c}$ and fringe amplitude ${B}_{l}^{c}$ ($1\le l\le 500)$ from the captured fringe patterns using Eqs. (11), (12) and (13), respectively;
(iii) Calculate the experimental variance of the phase of each pixel, ${\sigma }_{{\varphi }_{e}\left({u}^{c},{v}^{c}\right)}^{2}$, using Eq. (56) by merely replacing ${I}_{l}\left({u}^{c},{v}^{c}\right)$ by ${\varphi }_{{wl}}\left({u}^{c},{v}^{c}\right)$;
(iv) Calculate the means of the background intensity $\bar{{A}^{c}}\left({u}^{c},{v}^{c}\right)$ and fringe amplitude $\bar{{B}^{c}}\left({u}^{c},{v}^{c}\right)$ using Eq. (55) by merely replacing ${I}_{l}\left({u}^{c},{v}^{c}\right)$ by ${A}_{l}^{c}\left({u}^{c},{v}^{c}\right)$ and ${B}_{l}^{c}\left({u}^{c},{v}^{c}\right)$, respectively;
(v) Obtain the model-based phase variances of each pixel, ${\sigma }_{{\varphi }_{r}^{M}\left({u}^{c},{v}^{c}\right)}^{2}$, from a particular model *M* in Eqs. (38), (39), or (42), where *M* takes *F*, *A* and *S* for **M**_P-F_, **M**_P-A_ and **M**_P-S_, respectively;
(vi) Take the phase variance difference (PVD) as ${\delta }^{M}\left({\sigma }_{\varphi \left({u}^{c},{v}^{c}\right)}^{2}\right)={{\sigma }_{{\varphi }_{e}\left({u}^{c},{v}^{c}\right)}^{2}-\sigma }_{{\varphi }_{r}^{M}\left({u}^{c},{v}^{c}\right)}^{2}$ and then calculate its mean for quantitative comparison purpose.

**Fig. 5:**
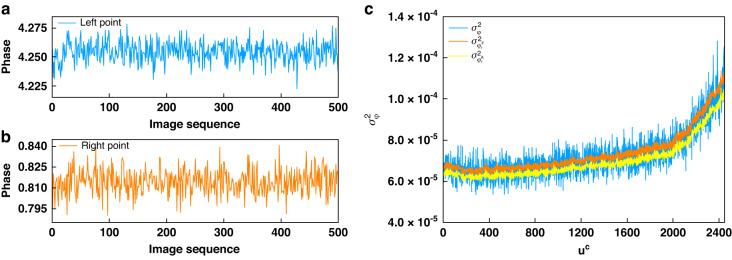
The temporal evolution of phase. **a** at Left point; **b** at Right point; **c** phase variance curves by measured, **M**_P-F_ and **M**_P-A_

**M**_P-F_**:** In Step (i), the ROI is the entire frame; In Step (ii), the evolutions of the calculated phases with respect to *l* for the same two pixels used in Validation of the projector noise are plotted in Fig. 5a, b, respectively; In Step (iii), the distribution of the experimental phase variances (in blue) is shown in Fig. 5c; In step (iv), the intensity of $\bar{{A}^{c}}\left({u}^{c},{v}^{c}\right)$ varies approximately from $63.92{\rm{DN}}$ to $109.74{\rm{DN}}$, while the intensity range of $\bar{{B}^{c}}\left({u}^{c},{v}^{c}\right)$ is approximately $56.06{\rm{DN}}$ to $96.96{\rm{DN}}$; In Step (v), $\bar{{A}^{c}}\left({u}^{c},{v}^{c}\right)$, $\bar{{B}^{c}}\left({u}^{c},{v}^{c}\right)$ and the overall gain $K=0.0232{\rm{DN}}{\left({{\rm{e}}}^{-}\right)}^{-1}$ are submitted into Eq. (38) to obtain the theoretical phase variance ${\sigma }_{{\varphi }_{r}^{F}\left({u}^{c},{v}^{c}\right)}^{2}$. The distribution of the middle row (in orange) is shown in Fig. 5c, which match very well with the blue line; In Step (vi), the mean of the PVD is calculated to be as small as −1.2269×10^−6^ which validates the proposed **M**_P-F_ where the phase error is introduced only by the camera noise.

**M**_P-A_**:** The validation is the same as for **M**_P-F_. In Step (vi), the theoretical result by **M**_P-A_ of the middle row is also plotted in Fig. 5c (in yellow), which, as expected, is slightly shifted downwards in relation to the orange line. Despite this, the result of **M**_P-A_ still matches the actual measurement result very well. The mean of the PVD is increased slightly to 2.7303 × 10^−6^, indicating that our approximation is valid.

**M**_P-S_**:** As assumed in this model, the fringe intensity is adjusted to be close to saturation and ${A}^{c}$ and ${B}^{c}$ in the captured fringe patterns are approximately equal. To achieve it, in Step (i), we slightly increase the aperture of the lens and use fringes with a period of 912 pixels. The ROI is changed from entire frame to a central area 40×50 pixels, which is reasonable as we only concern the precision limit; In Step (iv), in the ROI, $\bar{{A}^{c}}$ and $\bar{{B}^{c}}$ vary from $122.57{\rm{DN}}$ to $132.63{\rm{DN}}$ and from $117.61{\rm{DN}}$ to $127.26{\rm{DN}}$, respectively, thus both ${A}^{c}$ and ${B}^{c}$ have higher values and $\bar{{A}^{c}}\approx \bar{{B}^{c}}$; In Step (v), we substitute the saturation capacity measured in Validation of the camera noise model into Eq. (42) to obtain the phase variance; and in Step (vi), we obtain the mean of PVD to be 7.85 × 10^−7^, showing that **M**_P-S_ matches the experimental result well. It is worth highlighting that, for this model, we can skip Step (iv), and only require the saturation capacity to obtain the phase variance. Since the saturation capacity is usually provided by the manufacturer of the camera, this model is able to predict the phase variance successfully even without any measurements.

#### Comparison of all the phase noise models

In the above evaluations, two experiments were conducted. One may have noticed that, only the means of the PVDs are provided, instead of the means of the phase variances. This is because the phase variances in the first experiment are spatially varying, as clearly seen in Fig. 5c. However, within the small ROI (only 40×50 pixels) used in **M**_P-S_, such trend is ignorable. We thus re-calculate the means of the phase variances of all the models again and list them in Table 4 for a more direct comparison. Again, we validate that all the models agree well with their respective experimental results. More importantly, **M**_P-S_ perfectly predicts the lower limit of the phase variance without measurement (the measurement carried out here is only for validation purpose), providing a useful theoretical guideline for FPP system design.

**Table 4:** the mean of the phase variance within the ROI (40×50 pixels)

	Methods	Mean of phase variance
**First experiment**	**Experimental result**	$6.9215\times {10}^{-5}$
	**M**_P-F_	$7.0304\times {10}^{-5}$
	**M**_P-A_	$6.7004\times {10}^{-5}$
**Second experiment**	**Experimental result**	$4.3747\times {10}^{-5}$
	**M**_P-S_	$4.2962\times {10}^{-5}$

### Validation of the theoretical reconstruction precision of FPP

In this section, we verify the theoretical reconstruction precision using two FPP systems. The first FPP system has the following components, a projector (DLP6500), a camera (Model: MER2-502-79U3M-L) attached with a lens (Computar-M2512-MP2, 25 mm), with the following configuration: (i) the camera and the projector are positioned side by side so that ${t}_{1}^{p}$ is significantly larger than both ${t}_{2}^{p}$ and ${t}_{3}^{p}$; and (ii) the angle between the projector and the camera optical axes is approximately 7°. The FPP system is calibrated by Zhang’s method^14^ and the intrinsic and extrinsic matrices are consistent to the configuration,

57 $${A}^{c}=\left[\begin{array}{ccc}6959.443 & 0 & 1254.112\\ 0 & 6961.048 & 1056.854\\ 0 & 0 & 1\end{array}\right]$$

58 $${A}^{p}=\left[\begin{array}{ccc}3362.200 & 0 & 979.882\\ 0 & 3364.584 & 490.780\\ 0 & 0 & 1\end{array}\right]$$

59 $$\left[R^{p},{\bf{t}}^{p}\right]=\left[\begin{array}{ccc}0.993 & -0.005 & \begin{array}{cc}0.116 & -111.616\end{array}\\ -0.003 & 0.998 & \begin{array}{cc}0.068 & -49.472\end{array}\\ -0.116 & -0.067 & \begin{array}{cc}0.991 & -15.191\end{array}\end{array}\right]$$

A ceramic plate with a size of 300 × 300 mm^*2*^ and the peak-valley difference less than 0.005 mm is used as the measured object. The plate is placed at 14 different positions and measured one after another. In order to reveal possible spatial variation of the system performance, two ROIs are defined for analysis, one at the center of the image (center ROI) and the other near the lower-left corner of the image (corner ROI). The sizes of both ROIs are limited to 100 × 100 pixels. The fringe projection, fringe capturing, and phase calculation follow Validation of the phase noise models. The optimal three-frequency method^49^ is adopted for phase retrieval and unwrapping, where the fringe periods from low to high are ${T}_{0}=21$ pixels, ${T}_{1}=700/33$ pixels and ${T}_{2}=70/3$ pixels. Then ${u}^{p}$ is calculated by Eq. (16) and $\left({x}^{w},{y}^{w},{z}^{w}\right)$ is calculated from Eqs. (2), (3) and (7). For clarity, we denote the obtained 3D point cloud as $\left({x}_{0}^{c},{y}_{0}^{c},{z}_{0}^{c}\right)$. The point cloud data in each ROI are fitted into a plane as

60 $${A}_{0}{x}^{w}+{B}_{0}{y}^{w}+{C}_{0}{z}^{w}+{D}_{0}=0$$

**Fig. 6:**
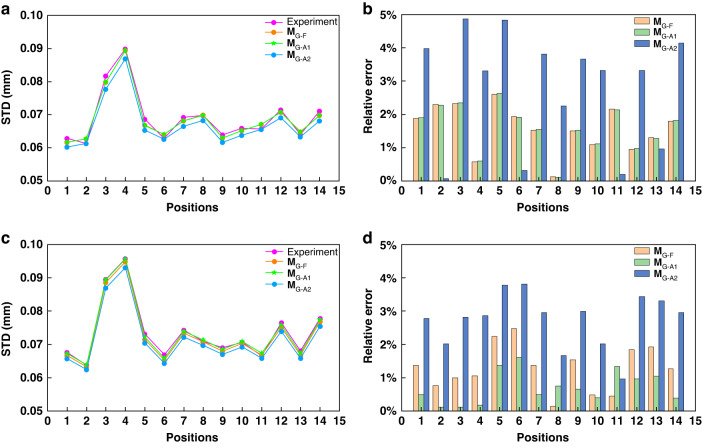
Precision comparison between the experiment results and three **M**_G_ models. **a**, **b** the STD distributions and the relative errors of the four methods at the center ROI; **c**, **d** the STD distributions and the relative errors of the four methods at corner ROI

The closest distance from each measured object point to the fitted plane is computed by

61 $$d\left({x}_{0}^{w},{y}_{0}^{w},{z}_{0}^{w}\right)=\frac{{A}_{0}{x}_{0}^{w}+{B}_{0}{y}_{0}^{w}+{C}_{0}{z}_{0}^{w}+{D}_{0}}{\sqrt{{A}_{0}^{2}+{B}_{0}^{2}+{C}_{0}^{2}}}$$

from which, the standard deviation (STD) is calculated as the experimental precision. We then move on to calculate their theoretical STDs in each ROI. Following the procedure described in Validation of the phase noise models, we obtain the theoretical phase variances, ${\sigma }_{{\varphi }_{r}^{F}\left({u}^{c},{v}^{c}\right)}^{2}$ of **M**_P-F_ and ${\sigma }_{{\varphi }_{r}^{A}\left({u}^{c},{v}^{c}\right)}^{2}$ of **M**_P-A_. Together with calibration parameters and ${z}_{0}^{w}\left({u}^{c},{v}^{c}\right)$, we further obtain ${\sigma }_{{z}^{F}\left({u}^{c},{v}^{c}\right)}$, ${\sigma }_{{z}^{A1}\left({u}^{c},{v}^{c}\right)}$, and ${\sigma }_{{z}^{A2}\left({u}^{c},{v}^{c}\right)}$ using our full model in Eq. (43), first approximation model in Eq. (44) and second approximation model in Eq. (45), respectively. Such calculation is repeated for all 14 plate positions. All these precision results in the center ROI and the corner ROI are shown in Fig. 6a, c, respectively, where the three theoretical precisions are seen to match the experimental precision very well. To see it more clearly, we also calculate the relative theoretical estimation errors against the experimental result for both the center and corner ROIs, which are shown in Fig. 6b and d, respectively. The relative errors of **M**_G-F_, **M**_G-A1_ and **M**_G-A2_ are less than 5% in both ROIs. We remark that the theoretical models agree well with the experimental result with little spatial variation in this experiment.

To further reveal the influence of different system configurations, the above validation is repeated using a second FPP system with the following modifications from the first one: (i) the projector model is changed from DLP6500 to DLP4500; (ii) the camera lens focal length is reduced from 25 mm (Computar-M2512-MP2) to 16 mm (Model: VTG-1614-M4); (iii) the imaging distance is almost halved; (iv) the positioning of the camera and projector exhibits noticeable deviations where ${t}_{1}^{p}$ is much reduced, ${t}_{2}^{p}$ is significantly increased, while ${t}_{3}^{p}$ is slightly increased. As a result, the angle between the camera and the projector optical axes is increased to 9°. Accordingly, the intrinsic and extrinsic matrices are obtained as,

62 $${A}^{c}=\left[\begin{array}{ccc}4683.754 & 0 & 1208.857\\ 0 & 4682.225 & 1035.941\\ 0 & 0 & 1\end{array}\right]$$

63 $${A}^{p}=\left[\begin{array}{ccc}1115.170 & 0 & 441.327\\ 0 & 2229.267 & 1162.533\\ 0 & 0 & 1\end{array}\right]$$

64 $$\left[ {R}^{p},{{\boldsymbol{t}}}^{p} \right] = \left[ \begin{array}{cccc} 0.990 & 0.069 & -0.120 & 78.093\\ -0.059 & 0.995 & 0.086 & -145.784\\ 0.126 & -0.078 & 0.989 & 23.508\end{array} \right]$$

**Fig. 7:**
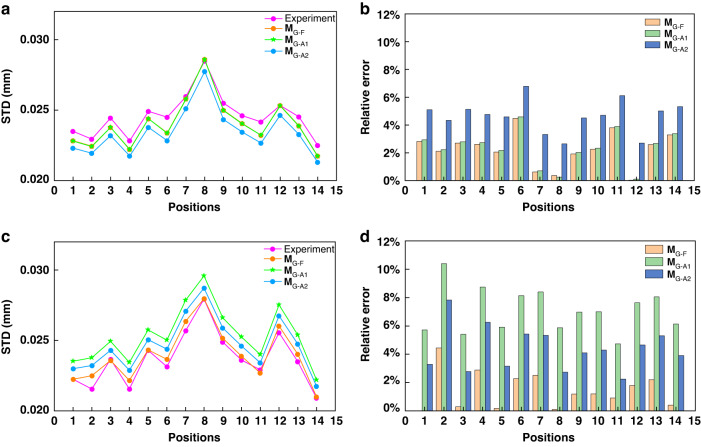
Precision comparison results between the experiment result and **M**_G_ models. **a**, **b** the STD distributions and the relative error of the four methods at center ROI; **c**, **d** the STD distributions and the relative error of the four methods at corner ROI

Note that, the camera is now almost above the projector, thus we project horizontal fringes^17^ and adjust both **M**_PtoG_ and ${E}_{r}$ expressions accordingly. All the results are shown in Fig. 7. We remark that (i) the full geometry model continues to provide good agreement with the experimental data with a relative error of less than 5%. Note that the seemingly larger discrepancy between the experimental and full geometry model results is larger in Fig. 7a, c than in Fig. 6a, c, which is actually due to the different scales of the vertical axes; (ii) however, the two approximation models present larger deviations, which is noticeably spatially varying. In the center ROI, the deviation of the first approximation model remained less than 5%, but in the corner ROI, it reaches 10%.

We also quantitatively investigate the approximation errors from the full geometry model (**M**_G-F_) to the first approximation model (**M**_G-A1_) and also from the first approximation model (**M**_G-A1_) to the second approximation model (**M**_G-A2_) for both ROIs in both experiments. The first approximation, ${E}_{r}$ is calculated from **M**_G-F_ and **M**_G-A1_ using Eq. (27) for all 14 positions and then calculate the average value. For the second approximation, ${E}_{r}$ is similarly calculated from **M**_G-A1_ and **M**_G-A2_. The results are given in Table 5, from which we observe that (i) the first approximation is dependent on both the pixel location and the system configuration, where the latter is more significant; and (ii) the second approximation interestingly gives a stable relative error of around 2.5%, which is because this approximation only simply neglects ${C}_{n}$. To summarize, if a system is reasonably well configured, then both approximation models, **M**_G-A1_ and **M**_G-A2_, can be used, with the latter preferred due to its simplicity. The study on the optimization of the FPP system configuration will be our future work.

**Table 5:** The relative errors of M_G-F_ and M_G-A1_ in both experiments

	From M_G-F_ to M_G-A1_		From M_G-A1_ to M_G-A2_	
	${E}_{r}$ in center ROI	${E}_{r}$ in corner ROI	${E}_{r}$ in center ROI	${E}_{r}$ in corner ROI
The first system	0.024%	0.890%	2.309%	2.413%
The second system	0.100%	5.718%	2.389%	2.521%

## Discussions

In this section, we discuss the following four aspects of an FPP system, which can help to improve the measurement precision.

### The thermal equilibrium of LEDs of the projector

**Fig. 8:**
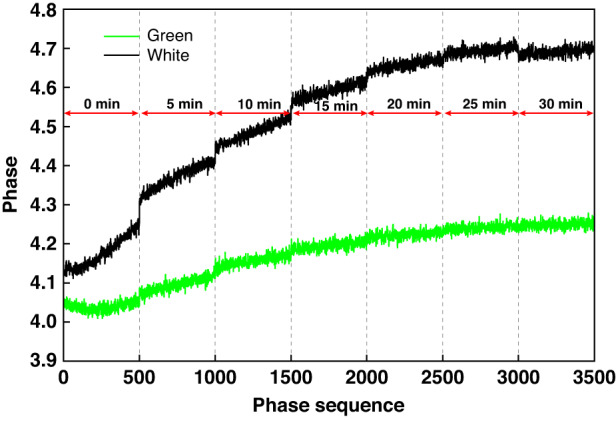
The phase performance of a randomly selected pixel on the camera after each pre-heating period

In our study, we find that the thermal equilibrium of LEDs of the projector is one of the main factors that affects the accuracy and precision of the measurement. We conduct seven pre-heating experiments for both green and white LED lights, increasing the pre-heating time from 0 min (i.e., no pre-heating) to 30 min with an increment of 5 min, which is indicated by the numbers above the red arrows in Fig. 8. After each pre-heating, we immediately acquire 500 sets of 9-step phase shifting fringe patterns, turn off the LED light and wait for it to cool down before starting the next pre-heating experiment. Figure 8 shows the phase value of a randomly selected pixel. The phase value increases before reaching the equilibrium. The stabilized phase values also differ between white and green LED lights, mainly because white light is composed of the red, green, and blue light. These phenomena suggests that pre-heating is necessary to achieve thermal equilibrium and subsequently a stable phase distribution; otherwise, both the accuracy and precision will be compromised.

### The optimal period of the projected fringe patterns

Equation (28) indicates that reducing the fringe period can enhance the measurement precision of FPP system. In the meantime, because of the influence of the system’s modulation transfer function (MTF),^50^ reducing the fringe period, i.e., increasing the fringe’s spatial frequency, will increase the phase variance. We investigate the impact of fringe period on the measurement precision by repeating the second experiment in Validation of the phase noise models with varying fringe periods. The result shows that the influence of the MTF is limited and a small period around *T* = 5 performance best in our focused FPP system. When the system is defocused, the influence of the MTF becomes more significant, and the optimal period will be increased to around *T* = 20.^34^

### The positioning of the measured object

The final precision of FPP, ${\sigma }_{z}$, is proportional to the square of the measurement distance, as shown in the **M**_PtoG_. Thus, reducing the measurement distance is the most straightforward and effective approach to improve the measurement precision. As a very rough estimation based on the experiment 5.4, if the measurement distance is reduced from the current 900 mm to about 240 mm, ${\sigma }_{z}$ can be reduced by 14 times from the current $70{\rm{\mu }}{\rm{m}}$ to $5{\rm{\mu }}{\rm{m}}$. Nevertheless, the field of view will be sacrificed.

### The correlation of the camera pixels

The simplest yet effective method for noise reduction is to combine neighboring 2 × 2 pixels into a super-pixel and take the average value as the intensity of this super-pixel. By assuming that the noises in these four pixels are independent, one would expect that the noise variance of the super-pixel will be reduced by four times compared to the original pixel, according to probability theory. However, our careful experiment which repeats the second experiment in Validation of the phase noise models demonstrates that the noise variance is reduced from $6.8669\times {10}^{-5}$ to $1.9495\times {10}^{-5}$ with a reduction of about 3.5 times. This surprising phenomenon is now easily explainable according to the diagram in Fig. 2 that the noises at neighboring pixels are not fully independent; for example, the quantization errors at neighboring pixels tend to be highly correlated. In our experiment, we obtain the following relationship for a super-pixel,

65 $${\sigma }_{I}^{2}=0.0954+0.0058I$$

By comparing it with Eq. (53), the overall gain *K* is reduced by four times, but ${C}_{n}$ almost remains the same. Such a finding is useful in understanding such pixel manipulations in practice.

## Conclusion

To characterize the measurement precision of an FPP system, in this paper, a complete precision model chain is proposed first, comprising four stage models (**M**_C_, **M**_I_, **M**_P_, and **M**_G_) and two transfer models (**M**_ItoP_ and **M**_PtoG_). Next, by moving forward from our previous study, we establish three phase-geometry transfer models (**M**_PtoG_) for different purposes. In addition, a non-Gaussian camera model **M**_C_ is adopted so that the noise can be properly estimated by simply referring to the known camera parameters. Together with the existing **M**_ItoP_, we derive all the other stage models, **M**_I_, **M**_P_ and **M**_G_, to fully understand the FPP performance. Among them, we highlight that we develop three phase noise models (**M**_P_) under different conditions so that readers can easily evaluate and compare the performance based on the measured phase, which has been a strong interest in many published research works; we then emphasize that we obtain four important 3D geometry noise models (**M**_G_), including a full model to accurately evaluate of the measurement precision, an approximation model that is suggested for use, and a precision limit model to guide the system design. Thus, the theoretical characterization of FPP is finally made possible. All our proposed models are experimentally validated.
